# Dietary DHA reduces downstream endocannabinoid and inflammatory gene expression and epididymal fat mass while improving aspects of glucose use in muscle in C57BL/6J mice

**DOI:** 10.1038/ijo.2015.135

**Published:** 2015-08-25

**Authors:** J Kim, M E Carlson, G A Kuchel, J W Newman, B A Watkins

**Affiliations:** 1 Lipid Chemistry and Molecular Biology Laboratory, Center on Aging, University of Connecticut Health Center, Farmington, CT, USA; 2 Genomics Institute of the Novartis Research Foundation, La Jolla, CA, USA; 3 Obesity and Metabolism Research Unit, USDA-ARS Western Human Nutrition Research Center, Davis, CA, USA; 4 Department of Nutrition, University of California, Davis, Davis, CA, USA; 5 West Coast Metabolomics Center, University of California, Davis, Davis, CA, USA

## Abstract

**Objectives:**

Endocannabinoid system (ECS) overactivation is associated with increased adiposity and likely contributes to type 2 diabetes risk. Elevated tissue cannabinoid receptor 1 (CB1) and circulating endocannabinoids (ECs) derived from the n-6 polyunsaturated acid (PUFA) arachidonic acid (AA) occur in obese and diabetic patients. Here we investigate whether the n-3 PUFA docosahexaenoic acid (DHA) in the diet can reduce ECS overactivation (that is, action of ligands, receptors and enzymes of EC synthesis and degradation) to influence glycemic control. This study targets the ECS tonal regulation of circulating glucose uptake by skeletal muscle as its primary end point.

**Design:**

Male C57BL/6J mice were fed a semipurified diet containing DHA or the control lipid. Serum, skeletal muscle, epididymal fat pads and liver were collected after 62 and 118 days of feeding. Metabolites, genes and gene products associated with the ECS, glucose uptake and metabolism and inflammatory status were measured.

**Results:**

Dietary DHA enrichment reduced epididymal fat pad mass and increased ECS-related genes, whereas it reduced downstream ECS activation markers, indicating that ECS activation was diminished. The mRNA of glucose-related genes and proteins elevated in mice fed the DHA diet with increases in DHA-derived and reductions in AA-derived EC and EC-like compounds. In addition, DHA feeding reduced plasma levels of various inflammatory cytokines, 5-lipoxygenase-dependent inflammatory mediators and the vasoconstrictive 20-HETE.

**Conclusions:**

This study provides evidence that DHA feeding altered ECS gene expression to reduce CB1 activation and reduce fat accretion. Furthermore, the DHA diet led to higher expression of genes associated with glucose use by muscle in mice, and reduced those associated with systemic inflammatory status.

## Introduction

Stimulation of the endocannabinoid system (ECS) has been known for some time to influence several physiological activities, including hunger, pain modulation, mood and inflammation, with the primary function appearing to affect energy homeostasis that is evident in the shift toward energy storage upon ECS activation.^1, 2, 3^ The receptors of the ECS, cannabinoid receptors 1 and 2 (CB1 and CB2), belong to the G_i/o_ protein-coupled receptor superfamily that can be activated by cannabinoid agonists or inhibited by antagonists with varying outcomes.^4^ Signal transduction following cannabinoid receptor ligand binding is generally characterized by marked inhibition of adenylyl cyclase.^5, 6, 7^

The ECS role in energy homeostasis is supported by multiple studies. Models using either pharmacological blockade of CB1- or CB1-null mice have shown reduced food intake.^8, 9^ However, lower energy intake did not account for the total body weight reduction.^10, 11^ In both obese subjects and leptin-deficient mice, an attenuated glucose uptake and fatty acid oxidation were reversed by CB1 antagonism.^12, 13^ Anandamide (AEA) and 2-arachidonoylglycerol (2-AG) are the two most frequently investigated endogenous ECS ligands or endocannabinoids (ECs). Levels of the two are elevated in obese compared with lean individuals.^14, 15^ Studies in animal models as well as in humans report that in conditions of obesity and hyperglycemia the ECS is in an overactivated state with ligands, receptors and enzymes of EC synthesis and degradation being upregulated.^14, 15, 16^

CB1 tissue expression and activity have been linked to glucose use and fatty acid oxidation in peripheral tissues, and intricately involved in obesity and excessive food intake.^17^ Both cannabinoid receptors are expressed in humans and rodents.^12^ Mice fed high-fat diets or subjected to diet-induced obesity overexpress CB1 in skeletal muscle,^10, 12^ a responsive tissue to ECS manipulation.^13^ For instance, leptin-deficient obese mice treated with the CB1 antagonist SR141716 for 7 days resulted in the induction of GLUT1-dependent glucose uptake in isolated soleus muscles.^13^ After SR141716 treatment, obese mice with elevated soleus muscle CB1 levels had corrected hyperglycemia, improved insulin resistance and reduced plasma insulin levels.^11^ SR141716 exposure increased 2-deoxyglucose uptake by the L6 skeletal muscle cell line.^18^ Skeletal muscle from human donors cultured with media previously used to culture adipocytes (that is, conditioned media) impaired insulin-stimulated Akt phosphorylation and reduced cellular glucose uptake.^19^ The adipocyte-conditioned media were found to contain AEA, 2-AG and several other ECs. Pretreatment of skeletal muscle cells with CB1 antagonists abolished the reduction of insulin-stimulated glucose uptake. Similarly, our group has shown higher levels of non-insulin-stimulated glucose uptake with the CB1 antagonist NESS0327 in proliferating and differentiated C2C12 myoblasts.^20^ Treatment with the docosahexaenoic acid (DHA)-derived EC-like compound, docosahexaenoyl ethanolamide (DHEA), also elevated glucose uptake. Collectively, these studies indicate that the ECS may directly modulate nutrient metabolism in skeletal muscle. Despite these advances, the molecular mechanisms underlying the actions of CB1 in skeletal muscle remain to be elucidated along with how other ECs as well as EC-like compounds derived from DHA influence receptor level and gene expression in muscle *in vivo*.

A dysregulated ECS is depicted in both the obese and insulin-resistant states with specific consequences on systemic glucose and fatty acid metabolism. Antagonizing CB1 is a potential target to ameliorate and correct conditions of hyperglycemia. One approach to study the ECS is to measure mRNA for ECS receptors and enzymes for the synthesis and degradation of EC and EC-like compounds *in vivo*. The mRNA levels of CB1 and enzymes for the synthesis and degradation of AEA and 2-AG were found to be increased in abdominal subcutaneous adipose tissue of obese compared with lean subjects.^21^ In contrast, the ECS is stimulated by exercise, leading to elevated plasma AEA and reductions in adipose CB1 expression.^22, 23^ These findings suggest that fat catabolism in adipose may result from activation of the ECS, and perhaps mobilization of fatty acids in muscle under these conditions may provide arachidonate for the synthesis of AEA and 2-AG. The arachidonate-derived EC support feeding and in excess contribute to detrimental consequences on energy homeostasis when their levels are elevated. The docosahexaenoate-derived EC-like compounds may offset the effects of arachidonate and AEA on glucose metabolism *in vivo* based on our recent findings demonstrating improvements in glucose uptake in C2C12 myoblasts.^20^ The n-3 PUFA have also demonstrated the ability to lower AEA and 2-AG^24^ and inflammatory molecules.^25^ This in turn could alter the ECS tone (action of ligands, receptors and the synthesis and degradation enzymes of the ECS) depending on the relative potency of the resulting EC and EC-like ligand availability within tissues.

The aim of the study was to determine how dietary DHA affects the ECS and glucose utilization in mice. The specific research hypothesis is that dietary DHA administered in a semipurified diet fed to C57BL/6J mice will restore EC tone to improve muscle glucose use and decrease fat accretion. Measurements of growth, body composition and tissue fatty acids along with ECs and an extensive array of inflammatory mediators and oxidative stress markers were performed to allow for the assessment of interactions between these fundamentally linked systems. Tissues were collected for quantitative real-time PCR and western blot analysis of EC enzymes related to glucose homeostasis to test our hypothesis.

## Materials and methods

### Animals, diets and experimental design

A total of 80 21-day-old male C57BL/6J mice (The Jackson Laboratory, Bar Harbor, ME, USA) were fed a modified AIN-93G diet (containing 11.04% fat) (Dyets, Bethlehem, PA, USA) for 62 or 118 days.^26^ Mice were assigned to either the control diet containing safflower oil or the DHA-enriched diet. Both diets were isocaloric and isonitrogenous, and thus the two diets varied only in their lipid source (Supplementary Table 1). Total fatty acid analysis of the diets showed that the control diet contained 0.2% n-3 polyunsaturated acid (PUFA) of total fatty acids and had a ratio of n-6/n-3 PUFA of 298, whereas the DHA diet contained 5.9% DHA of total fatty acids and had a ratio of n-6/n-3 of 10.5. The amount of DHA administered in the DHA diet was ∼1100 mg kg^−1^ per day that was determined by metabolic body size with an average daily food intake of 5 g per day per mouse for both groups.

Body weights were recorded once a week for the duration of the study, and food intake was recorded once each week. After 62 and 118 days of feeding, 9 mice from each group were weighed and anesthetized with isoflurane after fasting for 8 h. Blood was then collected for sera prep followed by cervical dislocation. The mice were then subjected to dual-energy X-ray absorptiometry (DXA) to measure lean and fat mass (pDXA Sabre; Norland Medical Systems, Inc., Fort Atkinson, WI, USA). Following the DXA scan, quadriceps, gastrocnemius, anterior tibialis, epididymal fat pads and liver were harvested, weighed and immediately frozen in liquid nitrogen. Animal care protocols were approved by and met all University of Connecticut Health Center Animal Care Committee procedures and guidelines.

### Dual-energy X-ray absorptiometry

Mouse lean mass (muscle mass density) and fat mass measurements were performed by DXA following the manufacturer's calibration protocols.^26^ Calibration was performed with a defined standard containing hydroxyapatite crystals embedded in Lucite (corresponding to bone mineral density=0.0594 g cm^−2^, percent fat=12.4%). Measurements were performed at a speed of 7 mm s^−1^ and a resolution of 0.5 × 0.5 mm.

### Fatty acid methyl ester analysis

Fatty acid composition of serum, anterior tibialis, quadriceps, epididymal fat pad and liver was measured by gas chromatography and flame ionization detection of fatty acid methyl esters. Tissue lipids were extracted with chloroform/methanol (2:1, vol/vol) and sonication. Extracted lipids were hydrolyzed with 0.5 N methanolic NaOH, and fatty acids were methylated with 10% (w/w) methanolic boron trifluoride. The solvent was removed, and the residue was reconstituted in isooctane and analyzed on a 7890A gas chromatograph equipped with a 30 m × 0.25 mm × 0.15 mm DB-225 column and flame ionization detector (Agilent Technologies, Palo Alto, CA, USA).^27^ Sample peaks were identified by comparison with authentic standards (Nu-Chek-Prep Inc., Elysian, MN, USA). Results of fatty acid methyl ester analysis values were determined by weight percentage reports based on the retention times and peak responses of authentic standards (not detected less than 50 ng per peak).

### EC and oxylipin analyses

ECs and oxylipins were measured in 36 serum samples (control and DHA-diet fed groups collected at day 62 and day 118) using 250 μl of serum as previously described.^28^ Briefly, the plasma samples were thawed on ice, added to solid-phase extraction column cartridges on a vacuum manifold, spiked with deuterated EC and oxylipin internal standards, up-diluted to 20% MeOH/0.1% acetic acid and gravity loaded onto 60 mg Oasis-HLB solid-phase extraction column (Waters, Inc., Milford, MA, USA) followed by vacuum. Columns were then wetted with 0.2 ml MeOH and eluted with 1.5 ml ethyl acetate by gravity. Solvent was removed by vacuum, sample reconstituted in 50 μl MeOH containing the internal standard 1-cyclohexyl-3-dodecyl-urea (Sigma Aldrich, St Louis, MO, USA) and samples were filtered and analyzed by ultraperformance liquid chromatography electrospray ionization-tandem mass spectrometry by back-to-back (+)-mode/(−)-mode injections for EC and oxylipin levels, respectively.^29,30^

### Quantitative real-time PCR

The primers sequences used in this work are shown in Supplementary Table 2. Additional details of gene expression are outlined in Supplementary Materials and Methods.

### Western blot analysis

Antibodies (α-mouse-CB1, α-mouse-CB2, α-rabbit-GLUT4, α-rabbit-Insulin-R) were purchased from Abcam (Cambridge, MA, USA) and detected via chemiluminescence. Additional details of protein expression analysis are outlined in Supplementary Materials and Methods.

### Statistical analysis

All data were analyzed for significance in SAS version 9.3 (SAS Institute Inc., Cary, NC, USA). Mean differences were evaluated by Student's *t*-test for gene expression or by Mann–Whitney *U*-test for EC and oxylipin concentrations. Significance level was defined as *P*<0.05. In the case for gene expression data where significant differences were found, Tukey's multiple comparison test was performed at a probability of α 0.05. Relative CT amounts for mRNA values were calculated from the standard curve for each gene that were normalized to GAPDH expression afterwards.

## Results

### Body weight, food intake and body composition

All mice in either diet group gained weight steadily throughout the feeding study and no differences were observed (Supplementary Figure 1). Feed intake on a cage basis showed no difference in the amount of diet consumed and weight change, weight gain and feed intake, and feed to gain ratios were not different between the control and DHA groups.

Whole-body DXA scan for lean mass and fat mass revealed differences at 118 days of feeding (Supplementary Figure 2). Mice fed the DHA diet had a higher lean mass (17.59 vs 12.56 g, *P*=0.0092) and lower fat mass (14.61 vs 23.79 g, *P*=0.0049) at the 118 day time point compared with those fed the control diet. Similarly, mouse epididymal fat pad mass was higher in the control compared with the DHA group (left, 1.09 vs 0.66 g, *P*=.0004; right, 1.12 vs 0.63 g, *P*=.0003). Mouse total fat mass determined by DXA was consistent with the results for epididymal fat pad weights.

### Muscle, fat and liver fatty acid composition

Mice fed the DHA semipurified diet had higher n-3 PUFA and lower arachidonate levels in anterior tibialis, epididymal fat pads and liver compared with those given the control diet (Supplementary Tables 3–5). After 62 days of DHA diet feeding, the anterior tibialis of mice showed higher levels of 16:1t, 20:1n9, 20:5n3, 22:6n3, 24:0 and 24:1n9 compared with mice fed the control diet (Supplementary Table 3), and a reduced ratio of n-6/n-3 PUFA (DHA=0.950, Control=10.8; *P*=0.0001). After 118 days of feeding, anterior tibialis from mice fed the DHA diet had higher levels of 14:0, 15:0, 16:0, 17:0, 20:5n3, 22:5n3 and 22:6n3 (Supplementary Table 3), and again the ratio of n-6/n-3 PUFA was lower in mice fed the DHA diet compared with the control group (DHA=1.43; Control=27.4; *P*<0.0001). In epididymal fat pads, 62 days of dietary DHA enrichment increased the levels of 14:0, 17:0, 18:0 and 22:6n3 (Supplementary Table 4) and lowered the ratio of n-6/n-3 PUFA (DHA=26.8; Control=135; *P*=0.0029). After 118 days of feeding, these fat pads showed elevated 12:0, 14:0, 15:0, 16:1t, 17:0, 20:5n3, 22:5n3 and 22:6n3 with DHA feeding (Supplementary Table 4), and a similar reduction in the ratio of n-6/n-3 PUFA (DHA=31.2, Control=712; *P*<0.001). After 62 days of DHA feeding, 15:0, 16:0, 18:2n6, 18:3n3, 20:5n3, 22:6n3, and 24:1n9 were elevated in the liver (Supplementary Table 5), and the ratio of n-6/n-3 PUFA was lower in the DHA diet compared with the controls (DHA=3.01, Control=54.1; *P*=0.0047). After 118 days of feeding, liver from the mice fed the DHA diet had higher levels of 15:0, 17:0, 18:0, 18:2n6, 20:0, 20:5n3, 22:0, 22:5n3 and 22:6n3 (Supplementary Table 5).

### Plasma ECs and related compounds

Higher levels of plasma DHEA were found consistently in mice fed the DHA diet at 62 and 118 days compared with control mice at both time points (Table 1). Moreover, DHA feeding was accompanied by lower levels of the arachidonic acid (AA)-derived AEA, DEA, 1-AG and 2-AG, as well as other EC-like compounds including 1-OG, 2-OG, SEA, OEA and dihomo GLA-EA at both time points. These observations in plasma ECs follow the remodeling of fatty acid composition for anterior tibialis (Supplementary Table 3), epididymal fat pad (Supplementary Table 4) and liver (Supplementary Table 5).

### ECS-, glucose uptake- and inflammation-related expression

**Figure 1:**
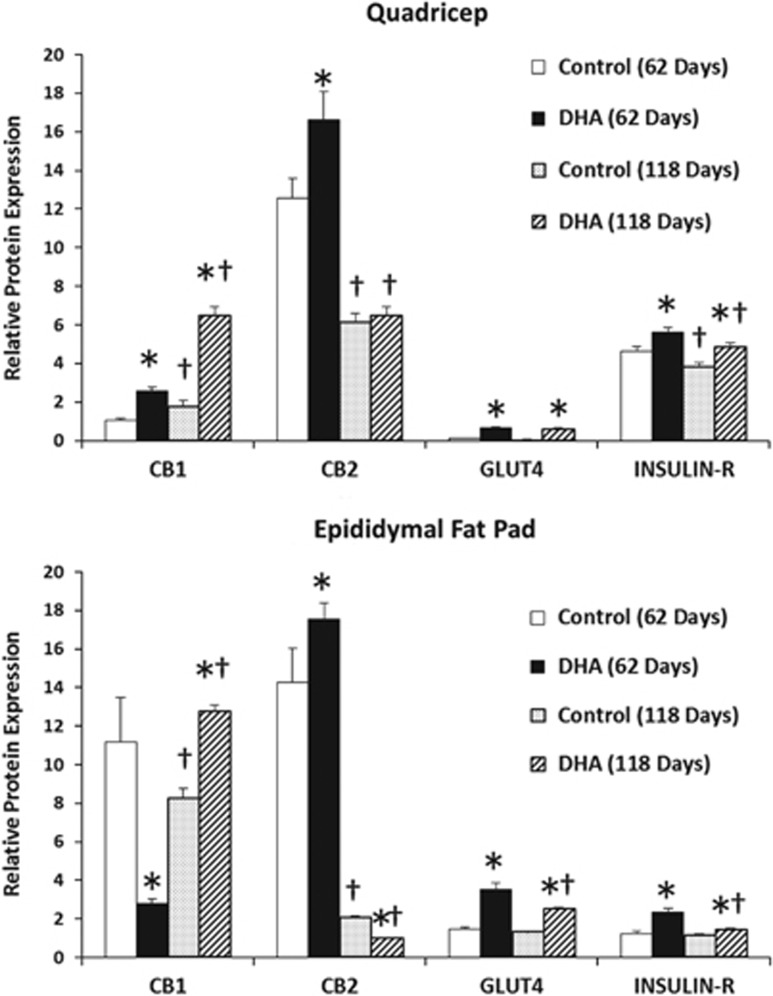
CB1, CB2, GLUT4 and INSULIN-R expression in mouse quadriceps and epididymal fat pad after 62 and 118 days of feeding AIN 93M semipurified diets: western blot comparison between the treatment (DHA) and control groups. Values are mean±s.d. for *n*=9 measurements. Differences between diet groups and time were compared by *t*-test. * represents differences between diet groups; ^†^ indicates significant difference over time within the same diet group.

Quadriceps and epididymal fat pad quantitative real-time PCR mRNA expression of ECS- and glucose metabolism-related genes revealed several differences. Reported values were normalized to the housekeeping gene *GAPDH* and shown in Tables 2 and 3. After 62 days of dietary DHA treatment, mRNA levels for both CB1 and CB2 in quadriceps were elevated. These changes were also maintained after 118 days of feeding. Western blot analysis followed by densitometry confirmed these findings (Figure 1). In contrast, DHA feeding reduced CB1 and increased CB2 mRNA in epididymal fat at 62 and 118 days, but protein levels matched mRNA results only at 62 days. CB1 protein increased whereas CB2 protein decreased after 118 days of DHA feeding.

As with the receptor levels, synthesis and degradation gene expression levels differed between the quadriceps muscle and adipose samples. The AEA synthesis enzyme, *N*-acyl phosphatidylethanolamine phospholipase D (NAPE-PLD), and the 2-AG synthesis enzyme, diacylglycerol lipase (DAGL)-α and DAGL-β, and fatty acid amide hydrolase (FAAH) enzyme responsible for the degradation of both ligands were elevated in quadriceps of mice fed the DHA diet after both 62 and 118 days of DHA diet feeding (Table 2). However, epididymal fat pads showed higher NAPE-PLD expression but lower levels of DAGL-α and -β isoforms at both 62 and 118 days of feeding, whereas FAAH was unchanged at 62 days but elevated at 118 days (Table 3).

### Cell status markers

Expression of myogenin and MyoD1 indicates myogenesis.^31^ Only after 62 days, mice fed the DHA diet showed a decrease in MyoD1 mRNA in quadriceps. The 5' adenosine monophosphate-activated protein kinase (AMPK)-α2 mRNA levels were higher in mice fed the DHA diet at both 62 and 118 days in both quadriceps and epididymal fat pads compared with the control mice (Tables 2 and 3).

### ECS confirmation (mitogen-activated protein kinase+adenylyl cyclase)

In order to validate ECS receptor activity, several downstream events found previously to occur with activation were analyzed.^4, 32, 33^ Here we see a higher expression of adenylyl cyclase and a lower expression of the mitogen-activated protein kinase enzymes, p42/p44, p38 and Jun N-terminal kinase, in the mice fed the DHA diet at both 62 and 118 days in quadriceps and epididymal fat pads. Though only mRNA expression was analyzed here, these findings in the DHA diet-fed mice demonstrate that the potential for downstream events of ECS activation are diminished.

### Glucose aspects

Quadriceps collected from mice fed the DHA diet had higher levels of Akt-1, insulin receptor, insulin receptor substrate-1, GLUT4 and GLUT1 mRNA after 62 and 118 days of feeding (Table 2). Western blot analysis reflected the higher levels of GLUT4 and insulin receptor expression (Figure 1). However, in epididymal fat pads DHA feeding reduced Akt-1, insulin receptor and GLUT4 mRNA, but increased insulin receptor substrate-1, GLUT1 and adiponectin mRNA (Table 3).

### Inflammation

DHA feeding lead to reductions in both tissue inflammatory cytokines and plasma pro-inflammatory lipid mediator profiles. Quadriceps from mice fed the DHA diet had lower levels of interleukin-6 and tumor necrosis factor-α mRNA after 62 and 118 days of feeding compared with the mice fed the control diet. Epididymal fat pad mRNA levels of interleukin-6, tumor necrosis factor-α and monocyte chemotactic protein-1 values were lower in mice fed the DHA diet after 62 and 118 days of feeding.

Several oxylipins were altered by DHA feeding (Table 4). Although many n-3 oxylipins were reduced and n-6 oxylipins increased by DHA feeding, not all detected compounds followed this behavior. For instance the AA-derived diols (DiHETrEs) and epoxides (EpETrEs) were reduced whereas the n-3 PUFA-derived HEPEs, HDoHEs, Di-HETEs, DiHDoPE, EpETEs and EpDoPEs were increased. In addition, by 118 days all of the measured 5-lipoxygenase-dependent metabolites were reduced including Lipoxin A4 (fivefold), LTB4 (twofold), 5-HETE (fourfold) and 5-KETE (sixfold; *P*=0.053). Of particular note, metabolites associated with 5-lipoxygenase metabolism including Lipoxin A4, LTB4 and 5-HETE were reduced, whereas the autooxidation markers 9-HETE, PGF2a and the measured fatty acid hydroperoxides were unchanged.

## Discussion

To date, few studies have directly investigated the interactions of n-3 fatty acid feeding and changes in the ECS as they relate to fat accretion and glucose metabolism. Here we specifically examined how dietary DHA affects the ECS and subsequent metabolic functions associated with obesity and type 2 diabetes in muscle and adipose tissues. Upon glucose challenge, skeletal muscle can account for as much as 95% of whole body glucose uptake.^34^ We have found that feeding mice a semipurified diet rich in DHA not only reduced the n-6/n-3 PUFA ratio in tissues, but also reduced epididymal fat pad mass and produced broad changes in ECS-associated gene and protein expression in both skeletal muscle and adipose tissues. These findings coincide with those in rodents fed n-6- or n-3-enriched diets in bone^26, 35, 36^ and quadriceps.^37^

As expected, changes in dietary fatty acids significantly altered tissue fatty acid composition and a number of tissue-specific processes associated with lipid metabolism. Interestingly, of the tissues evaluated here, the greatest increase in DHA and decrease in AA (20:4n6) was found in the anterior tibialis. Such changes in plasma membrane lipid composition can have direct consequence on the stability and architecture of membranes that in turn changes the availability of biosynthetic precursors and various membrane receptors, including those involved in glucose homeostasis. As shown in Supplementary Table 5, indices of stearoyl-coenzyme A desaturase activity (that is, 18:1n9/18:0 and 16:1n7/16:0) and of lipogenesis (that is, 16:0/18:2n6)^38^ in the liver were lower in the DHA group compared with the control diet-fed mice after 118 days of feeding. Such indices of steroyl-coenzyme A desaturase activity have previously been found to be elevated in individuals with abnormally high liver fatty acid synthesis^39^ and at risk for nonalcoholic fatty liver disease^40^ as well as insulin resistance.^41^ Moreover, these results are consistent with previous reports regarding the suppression of lipogenesis and steroyl-coenzyme A desaturase activity by n-3 feeding in mice,^42^ and may account for the reduced fat accumulation in the DHA group (Supplementary Table 5).

A considerable number of AA-derived oxylipins were lower in plasma, whereas those derived from n-3 PUFA were higher in mice fed the DHA diet. These findings are consistent with previous reports^43^ as numerous oxylipins showed changes paralleling shifts in tissue fatty acid composition (that is, increasing n-3 PUFA- and decreasing n-6 PUFA-derived oxylipins with DHA feeding) and large increases in eicosapentaenoic acid-derived metabolites are consistent with significant DHA retroconversion.^44^ Interestingly, of the numerous linoleate and α-linoleate-derived oxylipins measured, only 13-hydroxyoctadecadienoic acid was significantly changed by DHA feeding, with an approximately twofold increase after both 62 and 118 days. The implications of this change are unknown. The two- to six-fold reduction in classic 5-lipoxygenase-dependent pro-inflammatory mediators suggests a significant reduction in inflammatory tone, whereas the insignificant changes in PGF2α, 9-HETE and the measured fatty acid hydroperoxides of linoleate (HpODEs) and arachidonate (HpETEs) indicate that the different diets were not associated with significant differences in systemic oxidative stress.

Regarding plasma EC and EC-like compounds, the mice fed the DHA diet had reduced AEA and 2-AG but increased putative-cannabinoid receptor agonist DHEA. Reduction of the n-6/n-3 PUFA ratio and subsequent lowering of 2-AG has been observed in overweight and obese subjects given krill oil.^24^ An increase of circulating AEA and 2-AG has been reported in mice given dietary linoleic acid, leading to the development of diet-induced obesity.^45^ The n-3 PUFA-derived EC-like ligands have previously showed binding potential to cannabinoid receptors, with a greater affinity toward CB1 than CB2.^46, 47^ Although DHEA showed a lower affinity toward cannabinoid receptors than AEA, competition between these ligands still occurs, and the presence of DHEA may lower the likelihood of cannabinoid receptor activation. The classic CB2 receptor ligand, AEA, increases in humans with type 2 diabetes^28^ and regulates food intake in food-deprived mice, with peripheral AEA administration promoting hyperphagia in partially satiated mice.^48^ With the exception of palmatoylethanolamide, DHA feeding reduced the levels of all other measured acylethanolamides. Palmatoylethanolamide is an EC-like compound with peroxisome proliferator-activating receptor α-dependent anti-inflammatory effects.^49^ DHA feeding was generally associated with a decline in monoacylglycerides, and inverse correlations between this compound class and adiposity have been previously reported.^28^

In addition to the observed changes in circulating ECS ligands, expression of ECS-associated genes was also altered. The increase in mRNA levels of CB1, CB2, NAPE-PLD, DAGL-α, DAGL-β and FAAH in quadriceps of mice suggest an ever-increasing ability to turn over the ECS after 62 and 118 days of dietary DHA enrichment. Epididymal fat pads shared a similar response to muscle in mice fed the DHA diet as CB2 and NAPE-PLD were higher after 62 days, whereas CB2, NAPE-PLD and FAAH were higher after 118 days. As the DHA-derived DHEA is a weaker cannabinoid receptor ligand than AEA,^50^ an increase in these ECS-related mRNA levels could be a compensatory response.

The DHA-fed mice were found to have a lower potential for ECS activation, coinciding with an increase in adiponectin mRNA expression. The reduced expression of mitogen-activated protein kinase enzymes, p42/p44, p38 and Jun N-terminal kinase mRNA, and higher levels of adenylyl cyclase identified elsewhere,^4, 33^ are consistent with a reduction in ECS activation. Epididymal fat pad adiponectin mRNA expression was higher at both time points in mice fed the DHA diet. Adiponectin is an adipose-specific peptide hormone reported to reduce the expression of enzymes involved in lipogenesis and regulate glucose levels in humans^51^ and mice.^52^ CB1 activation by natural and synthetic ligands downregulates the expression and release of adiponectin.^14, 53^ Therefore, a decrease in CB1 agonist tone leading to an increase in adiponectin expression is consistent with the fat mass reduction observed in the DHA-fed mice.

AMPK mRNA was found to be higher in mice fed the DHA diet in both quadriceps and epididymal fat pads. AMPK is a known regulator of cellular energy metabolism, in which activation has been shown to inhibit lipogenesis.^54^ Although AMPK activation was not measured, elevated levels of mRNA indicate a lowered potential of lipid accumulation. Previously, AMPK activation has been found to improve glucose tolerance.^55^ Long-term administration of 5-aminoimidazole-4-carboxamide ribonucleoside, a drug that activates AMPK, to insulin-resistant Zucker rats was shown to improve glucose tolerance and reduce lipid accumulation. Others have shown that CB1 antagonism promoted an increase whereas activation led to a decrease in AMPK mRNA levels.^12^ Liver samples treated with Δ-9-tetrahydrocannabinol resulted in a decrease in AMPK activity.^56^ The results described in our study indicate that cannabinoid receptors are involved in metabolic processes related to glycolytic and oxidative flux for energy uptake and utilization in skeletal muscle. The impact on the metabolic regulatory machinery such as AMPK demonstrates a role of the ECS in fatty acid oxidation and glucose flux in skeletal muscle.

As shown in Figure 1, CB2 was found to be higher in epididymal fat pad of mice fed the DHA diet after 62 days. CB2 has been previously found to be involved in whole body glucose tolerance.^57^ When CB2 activation via specific agonists in rodents were concurrently treated with CB1 antagonism, non-insulin-stimulated glucose clearance was vastly improved. However, when CB2 was antagonized or CB1-specific agonists were administered, basal glucose clearance was impaired compared with the untreated cage mate. This study depicts CB1 and CB2 as having opposing effects with regard to glucose utilization.^57^ In the current study, higher levels of GLUT1 mRNA but lower levels of GLUT4, insulin receptor and Akt-1 in epididymal fat pads imply that DHA improved aspects of basal glucose uptake. Higher levels of GLUT1, GLUT4, Akt-1, insulin receptor and insulin receptor substrate-1 in quadriceps suggest that the potential for both insulin-stimulated and basal glucose uptake are improved with DHA feeding to mice. Despite possessing a lower affinity, DHEA can also bind to both cannabinoid receptors.^47^ Thus, higher levels of DHEA availability can result in competition with other ECs sharing affinity for the same receptors, possibly resulting in lower CB1 or CB2 activation. In addition, evidence of distinct downstream events after treatment with different ECs have been observed by our group such as greater uptake of glucose in proliferating and differentiated myoblast with DHEA compared with AEA or 2-AG.^20^ These findings could indicate a tissue-specific difference in how DHA may modulate the ECS that can ultimately influence metabolism of glucose.

While acknowledging the limitations of quantitative real-time PCR data, we believe that demonstrating mRNA measurements at two time points supported with DXA measurements of body fat is suggestive of an important relationship between dietary DHA and the ECS. However, this investigation would benefit from protein measurements of the genes examined. Future studies that include protein expression of AMPK, p42/44, p38 and Jun N-terminal kinase to validate downstream effectors of ECS activation will advance the understanding of dietary DHA.

**Figure 2:**
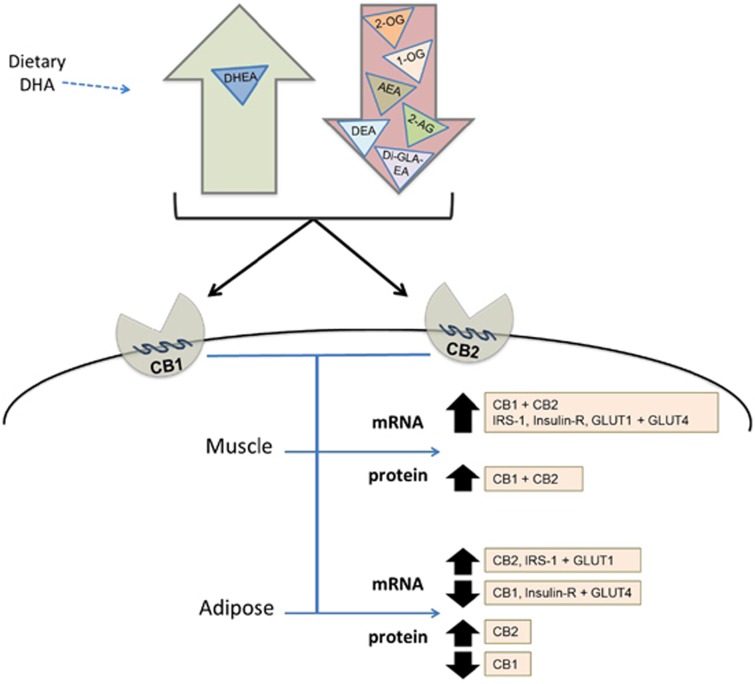
C57BL/6J mice fed a semipurified diet containing DHA showed higher levels of DHA and eicosapentaenoic acid (EPA) but lower AA in muscle and adipose tissues compared with those fed the control diet. Furthermore, the mice given DHA showed higher blood levels of DHEA and lower levels of AEA, 1-AG and 2-AG. The DHA diet also resulted in higher CB1 and CB2 protein levels and gene expression for these receptors in muscle compared with those given the control diet. Moreover, these changes resulting from DHA feeding also improved gene expression favorable for glucose uptake in muscle and lower glucose uptake by adipose associated with lower CB1 in mice.

Based on the findings of this investigation, DHA feeding to C57BL/6J mice led to compensatory increases of ECS-related mRNA levels and did not result in cannabinoid receptor activation. Mice fed the DHA diet were also found to have significantly less epididymal fat pad mass compared with control fed mice. Coupled with the increased mRNA levels of adiponectin and AMPK in epididymal fat pads, these findings suggest that a decrease in ECS activation can lead to an increase in glucose clearance and lipolysis in DHA-fed mice. It is likely that the increase in DHA-derived and decrease in AA-derived EC levels in mice fed the DHA diet are contributing factors for the results observed (Figure 2). Thus, the current investigation revealed that DHA can mediate ECS gene expression and positively influence the metabolism of systemic macromolecules.

## Figures and Tables

**Table 1:** Plasma endocannabinoid levels in mice fed the control and DHA semipurified diet after 62 and 118 days

*Metabolite*	*Substrate*	*Day 62*		*Day 118*		*Mean testing* P*-values*			
		*Control*	*DHA*	*Control*	*DHA*	*Day 62*	*Day 118*	*62 vs118 days control*	*62 vs 118 days DHA*
1-OG	18:1n9	322±30	172±19	514±61	213±12	‡	‡	‡	‡
2-OG	18:1n9	2730±158	15 820±184	3900±420	1880±91	‡	‡	‡	‡
1-LG	18:2n6	41.7±4.3	83.7±20.1	59.9±22.5	53.1±7.1	‡	0.4	*	†
2-LG	18:2n6	5870±179	5930±674	6350±513	5390±330	0.8	‡	‡	*
1-AG	20:4n6	5.01±0.89	1.96±0.35	7.22±1.71	1.77±0.16	‡	‡	†	0.2
2-AG	20:4n6	156±12	102±7	181±23	82.7±6.1	‡	‡	*	‡
PEA	16:0	23.4±1.4	26.1±2.1	32.4±42	35.4±2.1	†	0.07	‡	‡
SEA	18:0	460±34	258±29	838±73	470±45	‡	‡	‡	‡
OEA	18:1n9	312±47	187±27	846±215	347±67	‡	‡	‡	‡
LEA	18:2n6	27.0±2.7	21.3±2.4	42.5±3.58	29.0±2.2	‡	‡	‡	‡
α-LEA	18:3n3		0.02±0.00		0.02±0.01	—	—	—	1
Dihomo GLA EA	20:3n6	4.03±0.38	2.27±0.16	5.45±0.52	3.38±0.3	‡	‡	‡	‡
AEA	20:4n6	3.57±0.34	1.31±0.12	5.97±0.44	1.78±0.13	‡	‡	‡	‡
DEA	22:4n6	8.55±0.82	0.88±0.11	13.6±2.08	1.36±0.3	‡	‡	‡	‡
DHEA	22:6n3	0.3±0.03	3.41±0.32	0.27±0.02	4.23±0.31	‡	‡	*	‡

Abbreviations: AEA, anandamide; 1-AG, 1-arachidonoylglycerol; 2-AG, 2-arachidonoylglycerol; DEA, docosatetraenoyl ethanolamide; DHA, docosahexaenoic acid; DHEA, docosahexaenoyl ethanolamide; Dihomo-GLA-EA, dihomo-γ-linolenoyl ethanolamide; LEA, linoleoyl ethanolamide; α-LEA, α-linolenoyl ethanolamide; 1-LG, 1-linoleoyl ethanolamide; 2-LG, 2-linoleoyl ethanolamide; OEA, oleoyl ethanolamide; 1-OG, 1-oleoyl ethanolamide; 2-OG, 2-oleoyl ethanolamide; PEA, palmitoyl ethanolamide; SEA, stearoyl ethanolamide.

Values are mean±s.d. of nine samples from each group at each time point (values expressed as nM). Differences between diet groups and between time within the same diet groups were compared by Mann–Whitney *U*-test.

Significant differences in means: *P<0.05, ^†^*P*<0.01 and ^‡^*P*<0.001.

**Table 2:** Comparison of ECS and glucose uptake-related mRNA in mouse quadriceps after 62 and 118 days of feeding AIN 93M semipurified diets

*Measurements*	*Day 62*		*Day 118*		*Mean testing* P*-value*			
	*Control*	*DHA*	*Control*	*DHA*	*Day 62*	*Day 118*	*62 vs118 days control*	*62 vs 118 days DHA*
CB1	1.0±0.05	2.4±0.1	1.0±0.1	2.3±0.2	‡	‡	1	0.2
CB2	1.0±0.1	3.3±0.3	1.0±0.05	5.2±0.5	‡	‡	1	‡
NAPE-PLD	1.0±0.1	2.1±0.2	1.0±0.1	2.7±0.2	‡	‡	1	‡
FAAH	1.0±0.1	1.2±0.1	1.0±0.1	1.3±0.1	‡	†	1	0.06
DAGL-α	1.0±0.1	1.4±0.1	1.0±0.1	1.8±0.1	‡	‡	1	‡
DAGL-β	1.0±0.1	1.9±0.1	1.0±0.1	1.7±0.1	‡	‡	1	†
Akt-1	1.0±0.1	1.6±0.2	1.0±0.05	1.9±0.1	‡	‡	1	‡
Insulin R	1.0±0.1	1.9±0.2	1.0±0.05	4.1±0.3	‡	‡	1	‡
IRS-1	1.0±0.1	1.6±0.2	1.0±0.1	2.9±0.2	‡	‡	1	‡
GLUT4	1.0±0.1	2.7±0.1	1.0±0.04	4.1±0.4	‡	‡	0.9	‡
GLUT1	1.0±0.1	3.7±0.3	1.0±0.1	7.3±0.4	‡	‡	1	‡
Myogenin	1.0±0.03	1.0±0.1	1.0±0.04	1.0±0.03	0.8	0.06	1	0.2
MyoD1	1.0±0.04	0.9±0.1	1.0±0.1	1.0±0.1	*	0.4	0.9	0.3
IL-6	1.0±0.1	0.5±0.04	1.0±0.1	0.4±0.03	‡	‡	0.9	†
TNF-α	1.0±0.1	0.4±0.03	1.0±0.1	0.5±0.1	‡	‡	1	0.09
AMPK α2	1.0±0.1	1.4±0.1	1.0±0.1	1.8±0.1	‡	‡	1	‡
Adenylate Cyclase	1.0±0.1	1.1±0.1	1.0±0.1	1.3±0.1	*	‡	1	‡
p42/p44 (MAPK)	1.0±0.04	0.6±0.04	1.0±0.1	0.7±0.1	‡	‡	1	*
p38 (MAPK)	1.0±0.1	0.7±0.02	1.0±0.04	0.6±0.1	‡	‡	0.9	*
JNK (MAPK)	1.0±0.1	0.6±0.03	1.0±0.1	0.5±0.03	‡	‡	0.9	†

Abbreviations: Akt1, RAC-α serine/threonine-protein kinase (protein kinase B); CB1, cannabinoid receptor 1; CB2, cannabinoid receptor 2; DAGLα, diacylglycerol lipase-α DAGLβ, diacylglycerol lipase-β DHA, docosahexaenoic acid; EAC, endocannabinoid system; FAAH, fatty acid amide hydrolase; GLUT4, glucose transporter type 4; IL-6, interleukin-6; INS-R, insulin receptor; JNK, Jun N-terminal kinase; MAPK, mitogen-activated protein kinase; MyoD1, myogenic differentiation; NAPE-PLD, *N*-acyl phosphatidylethanolamine phospholipase D; TNF-α, tumor necrosis factor-α.

Values are the mean±s.d. (*n*=9) of ΔΔCT calculated from mouse tissue mRNA. Differences between diet groups and between time within the same diet groups were compared by *t*-test.

Mean values of quantitative real-time PCR (qPCR) data were compared by *t*-test using SAS software.

Significant differences in means: *P<0.05, ^†^*P*<0.01 and ^‡^*P*<0.001.

**Table 3:** Comparison of ECS and glucose uptake-related mRNA in mouse epididymal fat pad after 62 and 118 days of feeding AIN 93M semipurified diets

*Measurements*	*Day 62*		*Day 118*		*Mean testing* P*-value*			
	*Control*	*DHA*	*Control*	*DHA*	*Day 62*	*Day 118*	*62 vs118 days control*	*62 vs 118 days DHA*
CB1	1.0±0.1	0.4±0.03	1.0±0.1	0.2±0.01	‡	‡	0.9	‡
CB2	1.0±0.1	1.8±0.2	1.0±0.1	1.9±0.2	‡	‡	0.9	0.5
NAPE-PLD	1.0±0.1	2.1±0.2	1.0±0.1	2.4±0.2	‡	‡	1	†
FAAH	1.0±0.1	1.0±0.1	1.0±0.1	1.2±0.1	0.8	†		‡
DAGL-α	1.0±0.1	0.6±0.03	1.0±0.1	0.3±0.02	‡	‡	0.9	‡
DAGL-β	1.0±0.05	0.7±0.1	1.0±0.04	0.4±0.02	‡	‡	1	‡
Akt-1	1.0±0.1	0.7±0.04	1.0±0.1	0.4±0.01	‡	‡	1	‡
Insulin R	1.0±0.1	0.5±0.03	1.0±0.1	0.5±0.04	‡	‡	1	†
IRS-1	1.0±0.1	1.5±0.1	1.0±0.1	1.8±0.1	‡	‡	0.9	‡
GLUT4	1.0±0.1	0.6±0.04	1.0±0.1	0.3±0.02	‡	‡	1	‡
GLUT1	1.0±0.1	1.6±0.1	1.0±0.1	2.2±0.1	‡	‡	1	‡
Adiponectin	1.0±0.1	1.9±0.1	1.0±0.04	2.8±0.2	‡	‡	0.9	‡
IL-6	1.0±0.1	0.7±0.04	1.0±0.1	0.5±0.02	‡	‡	1	‡
TNF-α	1.0±0.05	0.5±0.04	1.0±0.1	0.5±0.03	‡	‡	1	‡
MCP1	1.0±0.1	0.7±0.05	1.0±0.1	0.6±0.03	‡	‡	1	‡
AMPK α2	1.0±0.1	1.6±0.1	1.0±0.1	1.6±0.1	‡	‡	1	0.4
Adenylate Cyclase	1.0±0.1	1.1±0.04	1.0±0.04	2.1±0.1	‡	‡	1	‡
p42/p44 (MAPK)	1.0±0.1	0.8±0.1	1.0±0.1	0.6±0.1	‡	‡	1	†
p38 (MAPK)	1.0±0.1	0.5±0.03	1.0±0.1	0.5±0.03	‡	‡	0.9	0.3
JNK (MAPK)	1.0±0.1	0.6±0.1	1.0±0.1	0.4±0.03	‡	‡	1	‡

Abbreviations: Akt1, RAC-α serine/threonine-protein kinase (protein kinase B); CB1, cannabinoid receptor 1; CB2, cannabinoid receptor 2; DAGLα, diacylglycerol lipase-α DAGLβ, diacylglycerol lipase-β DHA, docosahexaenoic acid; EAC, endocannabinoid system; FAAH, fatty acid amide hydrolase; GLUT4, glucose transporter type 4; IL-6, interleukin-6; INS-R, insulin receptor; JNK, Jun N-terminal kinase; MAPK, mitogen-activated protein kinase; MyoD1, myogenic differentiation; NAPE-PLD, *N*-acyl phosphatidylethanolamine phospholipase D; TNF-α, tumor necrosis factor-α.

Values are the mean±s.d. (*n*=9) of ΔΔCT calculated from mouse tissue mRNA. Differences between diet groups and between time within the same diet groups were compared by *t*-test.

Mean values of quantitative real-time PCR (qPCR) data were compared by *t*-test using SAS software.

Significant differences in means: *P<0.05, ^†^*P*<0.01 and ^‡^*P*<0.001.

**Table 4:** Plasma oxylipin levels in mice fed the control and DHA semipurified diet after 62 and 118 days

*Metabolites*	*Day 62*		*Day 118*		*Mean testing* P*-value*			
	*Control*	*DHA*	*Control*	*DHA*	*Day 62*	*Day 118*	*62 vs118 days control*	*62 vs 118 days DHA*
TXB2	14.6±6.6	12.3±5.2	52.5±30.3	14.3±4.0	0.8	0.2	0.2	0.8
6-keto PGF1a	1.53±0.64	1.06±0.59	1.91±0.82	1.02±1.00	0.6	0.5	0.7	1
PGE1	0.04±0.01	0.03±0.01	0.12±0.02	0.09±0.04	0.6	0.4	†	0.2
PGE2	0.52±0.12	0.35±0.15	1.18±0.38	0.51±0.17	0.4	0.1	0.1	0.5
PGD2	0.66±0.15	0.43±0.23	1.53±0.48	0.68±0.19	0.4	0.1	0.1	0.4
15-deoxy PGJ2	0.08±0.01	0.11±0.01	0.08±0.01	0.1±0.01	*	0.2	0.7	0.4
PGF2a	0.36±0.13	0.29±0.13	1.14±0.58	0.25±0.08	0.7	0.1	0.2	0.8
9,12,13-TriHOME	1.2±0.13	1.64±0.49	2.26±0.89	1.96±0.69	0.4	0.8	0.2	0.7
9,10-13-TriHOME	2.88±0.19	4.28±1.4	5.34±2.35	4.74±1.60	0.3	0.8	0.3	0.8
12,13-DiHOME	24.2±3.5	64.7±7.8	36.7±9.6	42.6±9.2	‡	0.6	0.2	0.07
9,10-DiHOME	193±22	307±31	264±73	215±42	†	0.5	0.3	0.08
15,16-DiHODE	0.31±0.04	0.48±0.05	0.94±0.51	0.39±0.05	*	0.3	0.2	0.2
12,13-DiHODE	0.03±0.01	0.05±0.01	0.09±0.04	0.03±0.01	0.2	0.2	0.2	0.3
9,10-DiHODE	0.09±0.01	0.10±0.01	0.56±0.24	0.11±0.03	0.7	0.09	0.08	0.6
14,15-DiHETrE	2.58±0.15	1.13±0.13	3.77±0.50	1.02±0.17	‡	‡	*	0.6
11,12-DiHETrE	1.13±0.08	0.48±0.06	2.02±0.34	0.49±0.09	‡	†	*	0.9
8,9-DiHETrE	2.21±0.11	0.62±0.03	2.90±0.39	0.69±0.09	‡	‡	0.1	0.5
5,6-DiHETrE	1.43±0.12	0.25±0.03	1.83±0.20	0.4±0.08	‡	‡	0.09	0.08
17,18-DiHETE	1.07±0.11	24.8±4.7	1.37±0.27	35.0±6.4	†	‡	0.3	0.2
14,15-DiHETE	0.17±0.02	3.99±0.95	0.23±0.05	4.99±1.10	†	†	0.2	0.5
19,20-DiHDoPE	0.77±0.04	20.0±2.2	1.02±0.23	25.3±3.7	‡	‡	0.3	0.2
Lipoxin A4	0.36±0.03	0.21±0.14	0.81±0.20	0.16±0.04	0.3	*	*	0.7
LTB4	0.18±0.05	0.19±0.12	0.39±0.08	0.20±0.04	1	*	*	1
6-trans-LTB4	0.23±0.07	0.28±0.22	0.57±0.16	0.19±0.05	0.8	*	0.07	0.7
5,15-DiHETE	0.03±0.01	0.03±0.01	0.05±0.01	0.02±0.01	0.9	0.2	0.2	0.9
13-HODE	235±24	576±133	513±120	887±296	*	0.2	*	0.3
9-HODE	25.8±2.2	64.3±18.2	46.3±12.7	62.4±16.5	0.06	0.4	0.1	0.9
13-HOTE	0.92±0.10	1.2±0.23	1.22±0.28	1.53±0.37	0.3	0.5	0.3	0.4
9-HOTE	0.45±0.03	0.75±0.15	0.60±0.13	0.78±0.17	0.06	0.4	0.3	0.9
15-HETrE	1.52±0.31	1.06±0.31	4.21±0.88	2.65±0.75	0.3	0.2	*	0.06
20-HETE	1.98±0.33	0.64±0.08	3.87±0.88	0.44±0.12	†	†	0.06	0.2
15-HETE	8.76±1.93	4.83±1.84	21.0±7.0	6.23±1.66	0.1	0.06	0.1	0.6
12-HETE	699±187	608±203	1820±0	1160±180	0.7	—	—	*
11-HETE	1.74±0.64	1.94±0.88	4.92±2.14	1.86±0.51	0.8	0.2	0.2	0.9
9-HETE	0.24±0.03	0.44±0.00	0.48±0.17	0.81±0.00	—	—	0.2	—
8-HETE	7.56±1.45	2.77±0.68	19.1±3.6	5.96±1.38	†	†	*	*
5-HETE	10.4±1.01	2.63±1.01	11.8±1.2	2.84±0.57	‡	‡	0.3	0.8
15-HEPE	0.21±0.03	1.28±0.29	0.24±0.03	3.19±0.81	†	†	0.5	*
12-HEPE	3.45±1.09	185±66	11.4±2.6	685±216	*	*	*	*
9-HEPE	0.01±0.00	0.47±0.09	0.10±0.09	0.74±0.18	†	†	0.3	0.2
5-HEPE	—	0.88±0.17	0.06±0.03	1.11±0.17	—	‡	—	0.3
17-HDoHE	1.48±0.32	21.2±5.3	2.9±0.60	79.9±21.8	†	†	*	*
14-HDoHE	12.1±3.3	277±87	28.0±8.3	1070±277	*	†	0.09	*
13-KODE	17.5±2.4	71.3±30.0	33.8±12.5	47.3±13.6	0.09	0.5	0.2	0.5
9-KODE	11.5±1.1	66.7±33	20.3±7.1	34.3±8.3	0.1	0.2	0.2	0.3
12(13)-Ep-9-KODE	11.1±1.2	70.8±41.5	24.0±10	34.8±11.3	0.2	0.5	0.2	0.4
5-KETE	1.4±0.2	0.81±0.67	2.22±0.84	0.38±0.19	0.4	0.05	0.3	0.5
12(13)-EpOME	102±6.2	188±18	171±24	150±19	‡	0.5	*	0.1
9(10)-EpOME	78.7±4.7	172±17	125±23	133±21	‡	0.8	0.07	0.1
15(16)-EpODE	0.86±0.11	1.79±0.48	2.0±0.60	1.17±0.12	0.08	0.2	0.08	0.2
12(13)-EpODE	0.12±0.01	0.24±0.03	0.25±0.06	0.22±0.03	†	0.6	*	0.6
9(10)-EpODE	0.88±0.11	2.0±0.7	2.02±0.70	1.30±0.22	0.1	0.32	0.1	0.3
14(15)-EpETrE	9.49±0.70	2.09±0.19	13.4±1.44	2.32±0.26	‡	‡	*	0.4
11(12)-EpETrE	21.8±1.5	5.57±0.76	28.4±3.1	5.39±0.54	‡	‡	0.07	0.8
8(9)-EpETrE	11.5±0.88	2.72±0.58	14.9±1.6	2.64±0.26	‡	‡	0.07	0.9
17(18)-EpETE	—	4.49±0.67	—	7.15±0.92	—	—	—	*
14(15)-EpETE	0.07±0.02	1.17±0.12	0.15±0.08	1.84±0.24	‡	‡	0.4	*
11(12)-EpETE	0.03±0.00	2.12±0.27	0.06±0.02	3.11±0.35	‡	‡	0.2	*
19(20)-EpDoPE	2.72±0.23	81.6±8.9	2.93±0.38	137±17	‡	‡	0.6	*
16(17)-EpDoPE	1.07±0.19	24.8±2.33	1.04±0.16	36.0±3.7	‡	‡	0.9	*
13-HpODE screen	0.01±0.00	0.07±0.04	0.03±0.01	0.03±0.01	0.1	1	0.3	0.3
9-HpODE screen	0.09±0.02	0.73±0.44	0.21±0.13	0.25±0.06	0.2	0.8	0.3	0.3
15-HpETE screen	0.03±0.01	0.03±0.02	0.06±0.02	0.01±0.00	0.8	0.09	0.3	0.4
12-HpETE screen	0.08±0.03	0.01±0.00	0.12±0.07	0.04±0.02	*	0.2	0.6	0.2

Abbreviations: CYP, cytochrome *P*450 superfamily; DHA, docosahexaenoic acid; LOX, lipoxygenase pathway; sEH, soluble epoxide hydrolase.

**LOX**: 5-HEPE, 5-hydroxy-6E,8Z,11Z,14Z,17Z-eicosapentaenoic acid; 12-HEPE, 12-hydroxy-5Z,8Z,10E,14Z,17Z-eicosapentaenoic acid; 5-HETE, 5-hydroxy-6E,8Z,11Z,14Z-eicosatetraenoic acid; 9-HETE, 9-hydroxy-5E,7Z,11Z,14Z-eicosatetraenoic acid; 12-HETE, 12-hydroxy-5E,8Z,10Z,14Z-eicosatetraenoic acid; 15-HEPE, 15-hydroxy-5Z,8Z,11Z,13E,17Z-eicosapentaenoic acid; 15-HETE, 15-hydroxy-15-hydroxy-5Z,8Z,11Z,13E-eicosatetraenoic acid; 17-HDoHE, 17-hydroxy-4Z,7Z,10Z,13Z,15E,19Z-docosahexaenoic acid.

**CYP**: 8(9)-EpETrE, 8(9)-epoxy-5Z,11Z,14Z-eicosatrienoic acid; 11(12)-EpETrE, 11(12)-epoxy-5Z,8Z,14Z-eicosatrienoic acid; 14(15)-EpETrE, 14(15)-epoxy-5Z,8Z,11Z-eicosatrienoic acid; 16(17)-EpDPE, 16(17)-epoxy-4Z,7Z,10Z,13Z,19Z-docosapentaenoic acid; 17(18)-EpETE, 17(18)-epoxy-5Z,8Z,11Z,14Z-eicosatetraenoic acid.

**sEH**: 5,6-DiHETrE, 5,6-dihydroxy-8Z,11Z,14Z-eicosatrienoic acid; 8,9-DiHETrE, 8,9-dihydroxy-5Z,11Z,14Z-eicosatrienoic acid; 11,12-DiHETrE-1,12-dihydroxy-5Z,8Z,14Z-eicosatrienoic acid; 14,15-DiHETE, 14,15-dihydroxy-5Z,8Z,11Z,17Z-eicosatetraenoic acid; 14,15-DiHETrE, 14,15-dihydroxy-5Z,8Z,11Z-eicosatrienoic acid; 17,18-DiHETE, 17,18-dihydroxy-5Z,8Z,11Z,14Z-eicosatetraenoic acid; 19,20-DiHDPA, 19,20-dihydroxy-4Z,7Z,10Z,13Z,16Z-docosapentaenoic acid.

Values are mean±s.d. of nine samples from each group at each time point (values expressed as nM). Differences between diet groups and between time within the same diet groups were compared by Mann–Whitney *U*-test.

Significant differences in means: *P<0.05, ^†^*P*<0.01 and ^‡^*P*<0.001.
