# Supplementary material for: Dietary DHA reduces downstream endocannabinoid and inflammatory gene expression and epididymal fat mass while improving aspects of glucose use in muscle in C57BL/6J mice
Source: Int J Obes (Lond). 2015 Aug 25;40(1):129–37. doi: 10.1038/ijo.2015.135 (PMC4722239; doi:10.1038/ijo.2015.135)
Supplement: Supplementary Information [file ijo2015135x1.docx]

**Supplemental Information**

Materials and Methods

***Quantitative real-time polymerase chain reaction (qPCR)***

100 mg of isolated adipose tissue or skeletal muscle were washed in PBS then homogenized in TRIzol (Invitrogen Corp., Carlsbad, CA, USA) reagent to isolate RNA which was then treated with DNase I (Ambion, Carlsbad, CA, USA) to remove DNA contamination. RNA (1 µg) was reverse transcribed to cDNA in a reaction mixture using RNA transcriptase superscript III (Invitrogen Corp., Carlsbad, CA, USA). Synthesized cDNA product was then used for quantitative RT-PCR. All samples were analyzed in triplicate. Fluorescence emission was detected and cycle threshold (CT) values were calculated in the linear range. Relative CT amounts were calculated from the standard curve for each gene, which were normalized to GAPDH expression afterwards.

***Western blot analysis***

Protein expression was analyzed by collecting whole cell lysates from quadriceps and epididymal fat pads after isolation. Approximately, 200 mg of both tissues were weighed and washed in cold PBS. Tissues were then lysed using RIPA buffer (50 mM Tris-HCl, pH 7.4 + 150 mM NaCl + 0.25% sodium deoxycholate + 1 mM EDTA) containing protease inhibitor (Calbiochem Protease inhibitor cocktail III; Merck KGaA, Darmstadt, Germany). Lysates were then centrifuged and supernatants were collected followed immediately by protein quantification using BCA (Thermo Scientific, Waltham, MA). Proteins were then separated by polyacrylamide gel electrophoresis and incubated with antibodies (α-mouse-CB1, α-mouse-CB2, α-rabbit-GLUT4, α-rabbit-Insulin-R, Abcam, PLC., Cambridge, MA, USA). Protein expression was detected using Westpico horseradish peroxidase chemiluminescence and imaged using a Chemidoc XRS+ system and Image Lab software (BioRad Laboratories, Hercules, CA, US).

Table S1. Fatty acid and ingredient composition of the semi-purified diets fed to mice

| Fatty acid (wt%) | Dietary treatments*^a,b,c,d^* | |
| --- | --- | --- |
|  | Control | DHA |
| 12:0 | 0.5 | 0.3 |
| 14:0 | 0.1 | 1.4 |
| 16:0 | 6.5 | 7.1 |
| 16:1n-7 | 0.1 | 0.3 |
| 18:0 | 2.3 | 2.1 |
| 18:1n-9 | 16.2 | 17.7 |
| 18:2n-6 | 72.9 | 63.6 |
| 18:3n-3 | 0.2 | 0.1 |
| 20:0 | 0.3 | 0.3 |
| 20:1n-9 | 0.2 | 0.2 |
| 22:0 | 0.3 | 0.3 |
| 22:1n-9 | 0.3 | 0.3 |
| 22:6n-3 | 0.0 | 5.9 |
| 24:1n-9 | 0.2 | 0.2 |
| SAT*^e^* | 9.7 | 11.7 |
| MONO | 16.8 | 18.5 |
| PUFA | 73.1 | 69.7 |
| n-6 PUFA | 72.9 | 63.6 |
| n-3 PUFA | 0.2 | 6.0 |
| n-6:n-3 | 364.5 | 10.6 |

*^a^*The semipurified basal diet contains the following (g/kg): casein, 200; corn starch, 367.076; DYETROSE, 122; sucrose, 100; cellulose, 50; L-lysine, 3; choline bitartrate, 2.5; salt mix, 35; vitamin mix, 10 (based on Watkins et al.[^26^](#_ENREF_26)).

*^b^*Salt mix provides (mg/kg diet): CaCO_3_, 12495; K_2_HPO_4_, 6860; C_6_H_5_O_7_K_3_·H_2_O, 2477; NaCl, 2590; K_2_SO_4_, 1631; MgO, 840; C_6_H_5_O_7_Fe, U.S.P., 212.1; ZnCO_3_, 57.75; MnCO_3_, 22.05; CuCO_3_, 10.5; KIO_3_, 0.35; Na_2_SeO_4_, 0.359; (NH_4_)_2_MoO_4_·H_2_O, 0.278; Na_2_O_3_Si·9H_2_O, 50.75; CrK(SO_4_)_2_·12H_2_O, 9.625; LiCl, 0.609; H_3_BO_3_, 2.853; NaF, 2.223; NiCO_3_, 1.113; NH_4_VO_3_, 0.231.

*^c^*Vitamin mix provides (mg/kg diet): thiamine HCl, 6; riboflavin, 6; pyridoxine HCl, 17; niacin, 30; calcium pantothenate, 16; folic acid, 2; biotin, 0.2; cyanocobalamin (B_12_) (0.1%), 25; vitamin A palmitate (500,000 IU/g), 8; vitamin E acetate (500 IU/g), 150; vitamin D_3_, 2.5; vitamin K_1_, 0.75.

*^d^*Dietary fat treatments includes safflower oil (Control) and 86.8% safflower oil + 13.2% DHASCO oil (DHA). Total fat content in each diet is 110.4 g/kg of diet.

*^e^*SAT, total saturated fatty acids; MONO, total monounsaturated fatty acids; PUFA, total polyunsaturated fatty acids.

Table S2. Primer sequences used for quantitative polymerase chain reaction (qPCR)

| Gene | Gene Symbol | Forward | Reverse |
| --- | --- | --- | --- |
| GAPDH | GAPDH | TGTGATGGGTGTGAACCACGAGAA | GAGCCCTTCCACAATGCCAAAGTT |
| CB1 | CNR1 | TCACACCTCAGAAGATGGCAAGGT | AGCAGATGATCAACACCACCAGGA |
| CB2 | CNR2 | TGAAGATCGGCAGTGTGACCATGA | AATGCTGAGAGGACCCACATGACA |
| NAPE-PLD | NAPEPLD | TGGCATTGTGCATGAAAGCCCTAC | AGTGGGCATGGTGTAGTTGTCAGT |
| FAAH | FAAH | TGGCATTGTGCATGAAAGCCCTAC | AGTGGGCATGGTGTAGTTGTCAGT |
| DAGL-α | DAGLA | CGACCACCAAGTGCAACCATTGAA | AACTCGGCGAATTCTAGCACCTGA |
| DAGL-β | DAGLB | TGTGTGTCAGCATGAGAGGAACCA | GTGGCGATGATGCCAATGACAACT |
| Akt-1 | AKT1 | GTAGCCATTGTGAAGGAG | TCTTGAGGAGGAAGTAGC |
| Insulin-R | INSR | TCCTGGAAATCGTCAACCTGCTCA | ACGATCCAACGGGACATTCTCCAT |
| IRS-1 | IRS1 | GGCACATCTCCTACCATT | CATCATCTCTGTATATTCCTCAAT |
| GLUT4 | GLUT4 | TCGTGGCCATATTTGGCTTTGTGG | TAAGGACCCATAGCATCCGCAACA |
| GLUT1 | GLUT1 | CATCGCCCTGGCCCTGCAGGAGC | GGCACCCCCCTGCCGGAAGCCGGA |
| Adiponectin | ADIPOQ | AATGACAGGAGCTGAAGGGC | AGGTGAAGAGAACGGCCTTG |
| Myogenin | MYOG | CGTGGGCATGTAAGGTGTGTAAGA | CATTCACTTTCTTGAGCCTGCGCT |
| MyoD1 | MYOD1 | TGAGCAAAGTGAATGAGGCCTTCG | AGAGCCTGCAGACCTTCGATGTA |
| IL-6 | IL6 | ATCCAGTTGCCTTCTTGGGACTGA | TAAGCCTCCGACTTGTGAAGTGGT |
| TNF-α | TNF | AGCCGATGGGTTGTACCTTGTCTA | TGAGATAGCAAATCGGCTGACGGT |
| MCP-1 | CCL2 | TGAGCCATGGGAACAAGGAAGTCT | TGTGCTGGTCTGTGATAGGCACAT |
| AMPK-α2 | PRKAA2 | TCCTGAAGACCCCTCCTACG | GAGTGGTTCTCAGCTGTGCT |
| Adenylyl cyclase | ADCY3 | GAAAGTGCGAACCCAGAGGA | ACTTGCGGACGTGTTCAGAT |
| p42/p44 (MAPK) | MAPK3 | AGCCACACGTTGGTACAGAG | CCAGAGCTTTGGAGTCAGCA |
| p38 (MAPK) | MAPK14 | GGCTGATGAGGAGATGACCG | ATGGGAGGCAGAGACTGGAT |
| JNK (MAPK) | MAPK8 | AGTGGGTTGCATCATGGGAG | CTGCATCTGAAGGCTGGTCT |

Figure S1. Weekly body weights (means ± SD) of mice for the duration of the study (P<0.05)


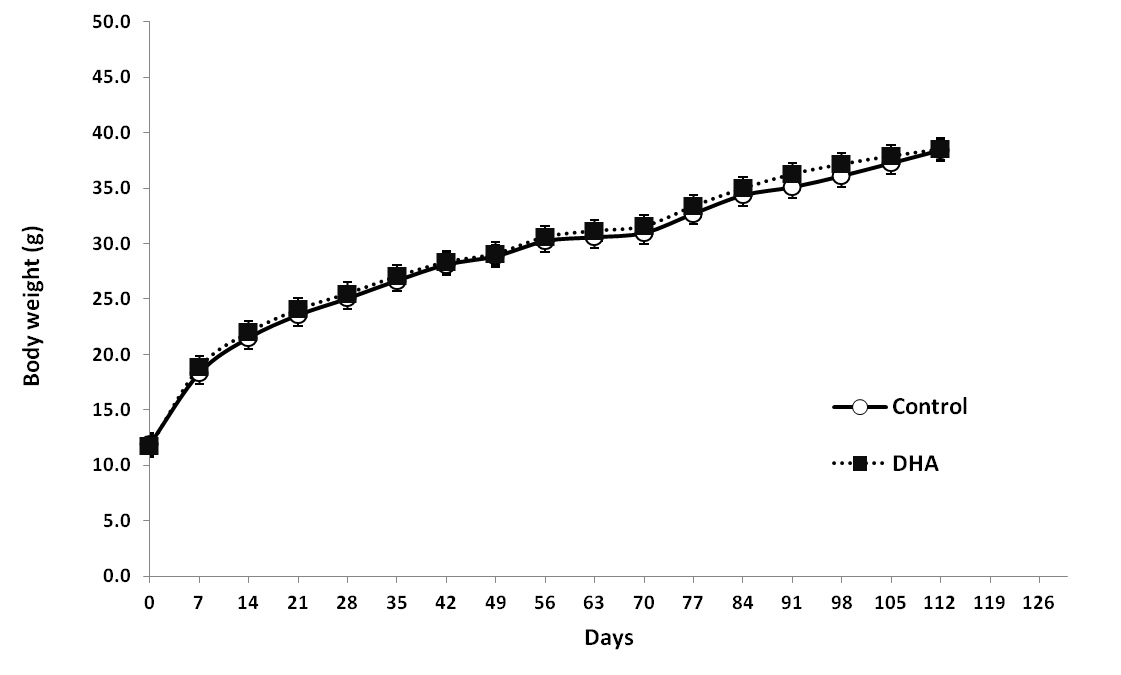


Figure S2. Mouse whole body DXA and epididymal fat pad weights at 62 and 118 d time points for the treatment (DHA) and control groups.


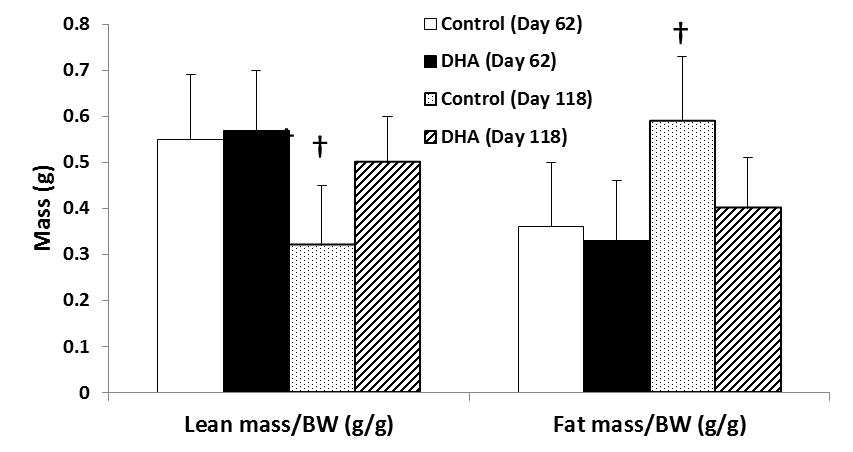


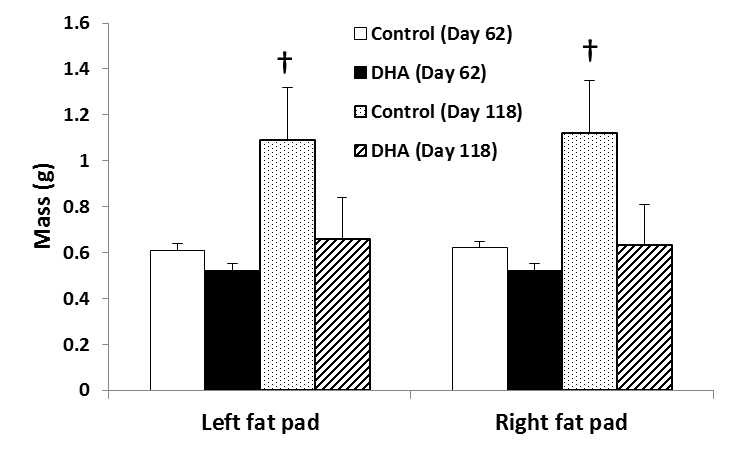


Values are means ± standard deviations for n = 9 measurements. Differences between diet groups and time were compared by t-test. † indicates significant difference compared to all other groups and time points.

Table S3. Fatty acid composition of total lipids in mouse muscle (anterior tibialis) after 62 and 118 d of feeding the semi-purified diets

| Fatty acid | D 62 | | |  | D 118 | | |  | Mean testing p-value | |
| --- | --- | --- | --- | --- | --- | --- | --- | --- | --- | --- |
|  | Control |  | DHA |  | Control |  | DHA |  | D 62 | D 118 |
| 12:0 | ND |  | ND |  | ND |  | 0.03 ± 0.05 |  |  | 0.08 |
| 14:0 | 0.80 ± 0.19 |  | 0.86 ± 0.18 |  | 0.99 ± 0.08 |  | 1.26 ± 0.15 |  | 0.5 | ‡ |
| 14:1n5 | 0.08 ± 0.09 |  | 0.03 ± 0.06 |  | 0.20 ± 0.03 |  | 0.19 ± 0.05 |  | 0.2 | 0.6 |
| 15:0 | 0.07 ± 0.06 |  | 0.08 ± 0.06 |  | 0.11 ± 0.01 |  | 0.13 ± 0.01 |  | 0.7 | ‡ |
| 16:0 | 18.75 ± 1.32 |  | 19.56 ± 0.91 |  | 16.74 ± 1.16 |  | 18.54 ± 1.10 |  | 0.2 | † |
| 16:1t | 0.58 ± 0.04 |  | 0.63 ± 0.02 |  | 0.63 ± 0.04 |  | 0.64 ± 0.03 |  | † | 0.41 |
| 16:1n7 | 5.09 ± 2.38 |  | 3.50 ± 1.32 |  | 8.83 ± 0.90 |  | 6.84 ± 1.34 |  | 0.1 | † |
| 17:0 | 0.14 ± 0.04 |  | 0.17 ± 0.03 |  | 0.09 ± 0.04 |  | 0.14 ± 0.02 |  | 0.2 | † |
| 18:0 | 8.39 ± 2.29 |  | 8.59 ± 1.80 |  | 4.73 ± 1.51 |  | 5.66 ± 1.27 |  | 0.8 | 0.2 |
| 18:1n9 | 11.38 ± 3.56 |  | 10.20 ± 4.21 |  | 17.17 ± 2.91 |  | 14.34 ± 2.02 |  | 0.5 | * |
| 18:1n7 | 2.35 ± 0.33 |  | 1.47 ± 0.57 |  | 2.25 ± 0.17 |  | 1.49 ± 0.06 |  | † | ‡ |
| 18:2n6 | 23.61 ± 5.20 |  | 21.07 ± 4.69 |  | 30.44 ± 3.94 |  | 25.66 ± 3.29 |  | 0.3 | * |
| 18:3n6 | 0.07 ± 0.08 |  | ND |  | 0.13 ± 0.05 |  | 0.03 ± 0.04 |  | * | ‡ |
| 18:3n3 | 0.05 ± 0.06 |  | 0.08 ± 0.07 |  | 0.01 ± 0.03 |  | ND |  | 0.4 | 0.4 |
| 20:0 | 0.01 ± 0.02 |  | ND |  | ND |  | ND |  | 0.4 |  |
| 20:1n9 | 0.24 ± 0.03 |  | 0.27 ± 0.03 |  | 0.24 ± 0.02 |  | 0.23 ± 0.02 |  | 0.1 | 0.3 |
| 20:2n6 | 0.47 ± 0.11 |  | 0.38 ± 0.07 |  | 0.28 ± 0.05 |  | 0.25 ± 0.04 |  | * | 0.2 |
| 20:3n6 | 0.74 ± 0.15 |  | 0.42 ± 0.05 |  | 0.55 ± 0.06 |  | 0.33 ± 0.03 |  | ‡ | ‡ |
| 20:4n6 | 10.40 ± 2.62 |  | 2.21 ± 0.42 |  | 5.85 ± 1.42 |  | 1.38 ± 0.20 |  | ‡ | ‡ |
| 20:5n3 | ND |  | 0.06 ± 0.06 |  | ND |  | 0.11 ± 0.05 |  | * | ‡ |
| 22:0 | ND |  | ND |  | ND |  | ND |  |  |  |
| 22:1n9 | ND |  | ND |  | ND |  | ND |  |  |  |
| 22:4n6 | 1.89 ± 0.49 |  | 0.01 ± 0.04 |  | 1.28 ± 0.28 |  | ND |  | ‡ | ‡ |
| 22:5n6 | 8.29 ± 2.69 |  | ND |  | 6.25 ± 2.27 |  | ND |  | ‡ | ‡ |
| 22:5n3 | 0.35 ± 0.10 |  | ND |  | 0.13 ± 0.03 |  | 0.39 ± 0.05 |  | ‡ | ‡ |
| 22:6n3 | 4.23 ± 1.38 |  | 27.03 ± 5.43 |  | 1.66 ± 0.63 |  | 20.14 ± 4.21 |  | ‡ | ‡ |
| 24:0 | 0.01 ± 0.02 |  | 0.12 ± 0.07 |  | ND |  | ND |  | † |  |
| 24:1n9 | 0.01 ± 0.02 |  | 0.16 ± 0.04 |  | 0.01 ± 0.04 |  | ND |  | ‡ | 0.4 |
| Saturated | 28.17 ± 3.34 |  | 29.37 ± 2.32 |  | 22.66 ± 2.54 |  | 25.77 ± 1.80 |  | 0.4 | † |
| Monounsaturated | 19.15 ± 5.82 |  | 15.63 ± 5.49 |  | 28.71 ± 3.56 |  | 23.10 ± 3.28 |  | 0.2 | † |
| PUFA | 50.11 ± 2.25 |  | 51.27 ± 1.46 |  | 46.59 ± 1.26 |  | 48.29 ± 1.54 |  | 0.2 | * |
| Total n-6 PUFA | 45.47 ± 0.93 |  | 24.09 ± 4.22 |  | 44.78 ± 1.05 |  | 27.65 ± 3.11 |  | ‡ | ‡ |
| Total n-3 PUFA | 4.64 ± 1.50 |  | 27.18 ± 5.43 |  | 1.80 ± 0.65 |  | 20.64 ± 4.23 |  | ‡ | ‡ |
| Ratio of n-6/n-3 | 10.80 ± 3.67 |  | 0.95 ± 0.36 |  | 27.39 ± 8.69 |  | 1.43 ± 0.48 |  | ‡ | ‡ |
| Area% | 98.01 ± 0.46 |  | 96.90 ± 3.02 |  | 98.59 ± 0.39 |  | 97.79 ± 0.46 |  | 0.3 | † |
| Total area | 11779 ± 4369 |  | 10131 ± 2070 |  | 5862 ± 2872 |  | 4857 ± 1746 |  | 0.3 | 0.4 |

Values are means ± standard deviations (n = 8 control and n = 9 DHA). ND = not detected. Differences between diet groups and time were compared by t-test. Significant differences in means are indicated at p<0.05 (*), p< 0.01 (†), and p<0.001 (‡).

Table S4. Fatty acid composition of total lipids in epididymal fat pad from mice after 62 and 118 d of feeding the semi-purified diets

| Fatty acid | D 62 | | |  | D 118 | | |  | Mean testing p-value | |
| --- | --- | --- | --- | --- | --- | --- | --- | --- | --- | --- |
|  | Control |  | DHA |  | Control |  | DHA |  | D 62 | D 118 |
| 12:0 | ND |  | ND |  | 0.03 ± 0.03 |  | 0.18± 0.02 |  |  | ‡ |
| 14:0 | 0.74 ± 0.06 |  | 1.28 ± 0.06 |  | 0.80 ± 0.17 |  | 1.26 ± 0.08 |  | ‡ | ‡ |
| 14:1n5 | ND |  | ND |  | 0.04 ± 0.06 |  | 0.04 ± 0.04 |  |  | 0.9 |
| 15:0 | 0.01 ± 0.04 |  | 0.01 ± 0.04 |  | 0.10 ± 0.01 |  | 0.13 ± 0.01 |  | 0.9 | ‡ |
| 16:0 | 16.1 ± 2.1 |  | 17.3 ± 2.20 |  | 15.6 ± 1.51 |  | 15.6 ± 1.6 |  | 0.3 | 0.9 |
| 16:1t | 0.53 ± 0.04 |  | 0.54 ± 0.09 |  | 0.55 ± 0.07 |  | 0.64 ± 0.08 |  | 0.9 | * |
| 16:1n7 | 4.15 ± 0.69 |  | 3.04 ± 0.86 |  | 4.88 ± 1.39 |  | 3.66 ± 0.87 |  | * | * |
| 17:0 | ND |  | 0.08 ± 0.07 |  | 0.09 ± 0.01 |  | 0.13 ± 0.01 |  | * | ‡ |
| 18:0 | 1.62 ± 0.26 |  | 2.05 ± 0.42 |  | 1.41 ± 0.25 |  | 1.59 ± 0.35 |  | * | 0.2 |
| 18:1n9 | 23.7 ± 0.81 |  | 22.6 ± 0.75 |  | 24.3 ± 1.72 |  | 23.5 ± 0.94 |  | * | 0.2 |
| 18:1n7 | 1.64 ± 0.21 |  | 1.01 ± 0.11 |  | 1.84 ± 0.31 |  | 1.07 ± 0.13 |  | ‡ | ‡ |
| 18:2n6 | 48.8 ± 2.4 |  | 48.8 ± 2.16 |  | 47.3 ± 2.3 |  | 48.9 ± 1.5 |  | 1 | 0.1 |
| 18:3n6 | 0.23 ± 0.05 |  | 0.05 ± 0.07 |  | 0.15 ± 0.03 |  | 0.12 ± 0.05 |  | ‡ | 0.3 |
| 18:3n3 | 0.02 ± 0.04 |  | ND |  | 0.07 ± 0.01 |  | 0.08 ± 0.01 |  | 0.3 | 0.06 |
| 20:0 | ND |  | ND |  | 0.04 ± 0.03 |  | 0.04 ± 0.03 |  |  | 1 |
| 20:1n9 | 0.44 ± 0.09 |  | 0.50 ± 0.07 |  | 0.42 ± 0.07 |  | 0.45 ± 0.05 |  | 0.2 | 0.3 |
| 20:2n6 | 0.21 ± 0.02 |  | 0.13 ± 0.05 |  | 0.19 ± 0.02 |  | 0.13 ± 0.01 |  | † | ‡ |
| 20:3n6 | 0.46 ± 0.03 |  | 0.23 ± 0.02 |  | 0.37 ± 0.07 |  | 0.20 ± 0.02 |  | ‡ | ‡ |
| 20:4n6 | 0.87 ± 0.13 |  | 0.20 ± 0.04 |  | 0.80 ± 0.09 |  | 0.15 ± 0.03 |  | ‡ | ‡ |
| 20:5n3 | ND |  | ND |  | 0.01 ± 0.02 |  | 0.10 ± 0.06 |  |  | ‡ |
| 22:0 | ND |  | ND |  | ND |  | ND |  |  |  |
| 22:1n9 | ND |  | ND |  | ND |  | ND |  |  |  |
| 22:4n6 | ND |  | ND |  | 0.17 ± 0.03 |  | ND |  |  | ‡ |
| 22:5n6 | ND |  | ND |  | 0.26 ± 0.03 |  | ND |  |  | ‡ |
| 22:5n3 | 0.14 ± 0.09 |  | 0.02 ± 0.05 |  | ND |  | 0.09 ± 0.02 |  | † | ‡ |
| 22:6n3 | 0.28 ± 0.06 |  | 1.93 ± 0.48 |  | ND |  | 1.39 ± 0.37 |  | ‡ | ‡ |
| 24:0 | ND |  | ND |  | ND |  | ND |  |  |  |
| 24:1n9 | ND |  | ND |  | ND |  | ND |  |  |  |
| Saturated | 18.5 ± 2.3 |  | 20.8 ± 2.6 |  | 18.0 ± 1.6 |  | 18.9 ± 1.9 |  | 0.07 | 0.3 |
| Monounsaturated | 30.0 ± 0.9 |  | 27.2 ± 1.4 |  | 31.5 ± 1.7 |  | 28.7 ± 1.6 |  | ‡ | † |
| PUFA | 51.0 ± 2.3 |  | 51.3 ± 1.8 |  | 49.3 ± 2.2 |  | 51.1 ± 1.5 |  | 0.7 | 0.06 |
| Total n-6 PUFA | 50.5 ± 2.4 |  | 49.4 ± 2.2 |  | 49.2 ± 2.2 |  | 49.5 ± 1.5 |  | 0.3 | 0.8 |
| Total n-3 PUFA | 0.43 ± 0.13 |  | 1.95 ± 0.52 |  | 0.07 ± 0.02 |  | 1.66 ± 0.37 |  | ‡ | ‡ |
| Ratio of n-6/n-3 | 135 ± 68 |  | 26.8 ± 6.5 |  | 712 ± 158 |  | 31.2 ± 6.8 |  | † | ‡ |
| Area% | 99.9 ± 0.3 |  | 99.8 ± 0.1 |  | 99.4 ± 0.1 |  | 99.4 ± 0.1 |  | 0.6 | 0.6 |
| Total area | 7360 ± 1570 |  | 7060 ± 1100 |  | 6750 ± 1270 |  | 7160 ± 2990 |  | 0.7 | 0.7 |

Values are means ± standard deviations (n = 8 control and n = 9 DHA). ND = not detected.

Differences between diet groups and time were compared by t-test. Significant differences in means are indicated at p<0.05 (*), p< 0.01 (†), and p<0.001 (‡).

Table S5. Fatty acid composition of lipids in liver of mice after 62 and 118 d of feeding the semi-purified diets

| Fatty acid | D 62 | | |  | D 118 | | |  | Mean testing p-value | |
| --- | --- | --- | --- | --- | --- | --- | --- | --- | --- | --- |
|  | Control |  | DHA |  | Control |  | DHA |  | D 62 | D 118 |
| 12:0 | 0.005 ± 0.01 |  | 0.03 ± 0.04 |  | ND |  | ND |  | 0.07 |  |
| 14:0 | 0.41± 0.17 |  | 0.51 ± 0.18 |  | 0.56 ± 0.10 |  | 0.39± 0.07 |  | 0.3 | ‡ |
| 14:1n5 | 0.01 ± 0.02 |  | 0.003 ± 0.01 |  | ND |  | ND |  | 0.8 |  |
| 15:0 | 0.02 ± 0.04 |  | 0.09 ± 0.05 |  | 0.07 ± 0.01 |  | 0.09 ± 0.01 |  | † | * |
| 16:0 | 21.0 ± 0.93 |  | 22.8 ± 0.67 |  | 23.7 ± 2.6 |  | 23.4 ± 0.61 |  | ‡ | 0.7 |
| 16:1t | 0.62 ± 0.24 |  | 0.59 ± 0.14 |  | 1.20 ± 0.36 |  | 0.70 ± 0.30 |  | 0.7 | † |
| 16:1n7 | 2.34 ± 1.1 |  | 1.54 ± 0.47 |  | 3.99 ± 0.60 |  | 2.16 ± 0.77 |  | 0.07 | ‡ |
| 17:0 | 0.18 ± 0.05 |  | 0.16 ± 0.03 |  | 0.10 ± 0.02 |  | 0.15 ± 0.03 |  | 0.3 | † |
| 18:0 | 10.0 ± 2.4 |  | 6.22 ± 1.67 |  | 4.34 ± 1.67 |  | 7.51 ± 1.89 |  | † | † |
| 18:1n9 | 14.2 ± 4.1 |  | 10.6 ± 2.4 |  | 27.5 ± 6.2 |  | 15.6 ± 6.20 |  | * | ‡ |
| 18:1n7 | 1.66 ± 0.77 |  | 0.93 ± 0.85 |  | 3.68 ± 0.77 |  | 1.50 ± 0.74 |  | 0.08 | ‡ |
| 18:2n6 | 26.7 ± 3.2 |  | 35.3 ± 3.5 |  | 21.5 ± 3.3 |  | 25.1 ± 3.0 |  | ‡ | * |
| 18:3n6 | 0.58 ± 0.15 |  | 0.66 ± 0.20 |  | 0.53 ± 0.10 |  | 0.28 ± 0.05 |  | 0.3 | ‡ |
| 18:3n3 | 0.01 ± 0.02 |  | 0.06 ± 0.04 |  | ND |  | ND |  | ‡ |  |
| 20:0 | 0.27 ± 0.08 |  | 0.10 ± 0.04 |  | 0.16 ± 0.08 |  | 0.25 ± 0.04 |  | ‡ | † |
| 20:1n9 | 0.46 ± 0.11 |  | 0.22 ± 0.02 |  | 0.68 ± 0.15 |  | 0.45 ± 0.20 |  | ‡ | * |
| 20:2n6 | 0.47 ± 0.07 |  | 0.24 ± 0.06 |  | 0.38 ± 0.11 |  | 0.33 ± 0.06 |  | ‡ | 0.3 |
| 20:3n6 | 1.25 ± 0.20 |  | 0.58 ± 0.17 |  | 1.24 ± 0.45 |  | 1.26 ± 0.24 |  | ‡ | 0.9 |
| 20:4n6 | 13.7 ± 4.8 |  | 4.35 ± 1.80 |  | 6.65 ± 3.03 |  | 5.52 ± 1.15 |  | ‡ | 0.3 |
| 20:5n3 | ND |  | 1.11 ± 0.20 |  | ND |  | 1.21 ± 0.33 |  | ‡ | ‡ |
| 22:0 | ND |  | 0.02 ± 0.04 |  | 0.01 ± 0.03 |  | 0.09 ± 0.05 |  | 0.1 | † |
| 22:1n9 | 0.004 ± 0.01 |  | ND |  | 0.02 ± 0.03 |  | 0.02 ± 0.03 |  | 0.4 | 1 |
| 22:4n6 | 0.83 ± 0.16 |  | 0.03 ± 0.03 |  | 0.67 ± 0.34 |  | 0.09 ± 0.05 |  | ‡ | ‡ |
| 22:5n6 | 2.95 ± 0.95 |  | 0.13 ± 0.20 |  | 1.68 ± 0.74 |  | ND |  | ‡ | ‡ |
| 22:5n3 | ND |  | 0.05 ± 0.14 |  | ND |  | 0.53 ± 0.05 |  | 0.4 | ‡ |
| 22:6n3 | 1.10 ± 0.47 |  | 12.8 ± 2.1 |  | 0.40 ± 0.18 |  | 12.7 ± 2.9 |  | ‡ | ‡ |
| 24:0 | ND |  | 0.01 ± 0.03 |  | ND |  | 0.02 ± 0.04 |  | 0.4 | 0.2 |
| 24:1n9 | ND |  | 0.11 ± 0.04 |  | ND |  | 0.01 ± 0.03 |  | ‡ | 0.4 |
| Saturated | 31.9 ± 1.7 |  | 30.0 ± 1.5 |  | 28.9 ± 2.9 |  | 31.8 ± 1.7 |  | * | * |
| Monounsaturated | 18.7 ± 5.3 |  | 13.4 ± 2.2 |  | 35.8 ± 7.5 |  | 19.8 ± 7.9 |  | * | ‡ |
| PUFA | 47.6 ± 5.2 |  | 55.3 ± 1.3 |  | 33.1 ± 7.6 |  | 47.1 ± 6.6 |  | † | ‡ |
| Total n-6 PUFA | 46.5 ± 4.8 |  | 41.3 ± 2.0 |  | 32.7 ± 7.5 |  | 32.6 ± 3.7 |  | * | 1 |
| Total n-3 PUFA | 1.10 ± 0.45 |  | 14.0 ± 2.1 |  | 0.40 ± 0.18 |  | 14.4 ± 3.2 |  | ‡ | ‡ |
| Ratio of n-6/n-3 | 54.1 ± 39.5 |  | 3.01 ± 0.55 |  | 90.4 ± 21.9 |  | 2.3 ± 0.4 |  | † | ‡ |
| Ratio of 18:1n9/18:0 | 0.11 ± 0.08 |  | 0.07 ± 0.05 |  | 0.17 ± 0.04 |  | 0.09 ± 0.03 |  | 0.2 | ‡ |
| Ratio of 16:1n7/16:0 | 1.41 ± 0.49 |  | 1.70 ± 0.51 |  | 6.33 ± 1.19 |  | 2.08 ± 0.28 |  | 0.3 | ‡ |
| Ratio of 18:2n6/16:0 | 0.79 ± 0.12 |  | 0.62 ± 0.13 |  | 1.10 ± 0.23 |  | 0.88 ± 0.10 |  | * | * |
| Area% | 98.8 ± 2.9 |  | 99.2 ± 0.6 |  | 99.0± 0.2 |  | 99.4 ± 0.1 |  | 0.6 | ‡ |
| Total area | 11600 ± 10400 |  | 17000 ± 8700 |  | 6460 ± 2420 |  | 3510 ± 1130 |  | 0.3 | ‡ |

Values are means ± standard deviations (n = 8 control and n = 9 DHA). ND = not detected.

Differences between diet groups and time were compared by t-test. Significant differences in means are indicated at p<0.05 (*), p< 0.01 (†), and p<0.001 (‡).
